# Novel method using DW-MRI and ADC images to guide stereotactic biopsy for the diagnosis small primary angiitis of the central nervous system: a case report

**DOI:** 10.1186/s40001-021-00529-3

**Published:** 2021-06-23

**Authors:** Xu Su, Liang Han, Mengxing Li, Zhengming Wang, Jiadui Gao, Yu Tian, Chao Du

**Affiliations:** 1 Department of Neurosurgery, China-Japan Union Hospital of Jilin University, No. 126, Xiantai Street, Changchun, 130033 China; 2 Department of Pathology, China-Japan Union Hospital of Jilin University, Changchun, China; 3 Beijing Neurosurgical Institute , Beijing, China

**Keywords:** Case report, Biopsy guiding method, DW-MRI/ADC map, Primary angiitis of the central nervous system, Diagnosis

## Abstract

**Objective:**

To determine the role of diffusion-weighted magnetic resonance imaging (DW-MRI) and apparent diffusion coefficient (ADC) imaging to guide stereotactic biopsy for the diagnosis of intracranial angiitis.

**Case presentation:**

In a 28-year-old woman who had experienced inactive headache and right limbs numbness for 4 days, preoperative magnetic resonance (MR) scanning, enhanced scanning, diffusion tensor imaging, magnetic resonance spectroscopy, diffusion-weighted imaging (DWI), and ADC image scanning were performed. Stereotactic biopsy was performed in one target where the area of edema detected with MR FLAIR, and two targets where the area shown as a high-value and a lower value area in the DWI/ADC image. Pathological examinations together with computed tomographic and enhanced MRI scans were conducted after surgery. A preoperative enhanced MRI scan showed a uniform low-intensity lesion in the patient’s left centrum semiovale, with a volume of 3.1 cm^3^. The DWI and ADC images showed uneven high-intensity signals and different ADC values in the lesion area, respectively. During surgery, tissues around the lesion and the lesion center were sampled at the three selected targets. The postoperative pathological diagnosis was primary angiitis of the central nervous system, and the patient was given anti-inflammatory medication and hormone therapy. The 3-year follow-up confirmed that the patient had recovered well, with a Glasgow Outcome Scale score of five.

**Conclusion:**

DW-MRI and ADC images can be reliably used to determine the location of small intracranial lesions, and guide stereotactic biopsy to facilitate the diagnosis of primary vasculitis of the central nervous system.

## Introduction

Primary angiitis of the central nervous system (PACNS) is an idiopathic inflammatory syndrome confined to the brain parenchyma, spinal cord, and leptomeninges, that mainly involves the vascular walls [1]. The incidence rate of PACNS is estimated to be 2–3 cases per 1 million individuals [2, 3]. A Mayo Clinic study of 163 patients with PACNS, published in 2015, is still the most extensive sample of the disease yet reported [4, 5]. The etiology of PACNS is not yet clear. The peak age of onset is around 50 years, and it may be slightly more prevalent in women than in men. It often affects young people who lack common risk factors for cerebrovascular disease [1, 2, 6]. The clinical manifestations of PACNS are diverse, and the specificity of laboratory tests and imaging is poor, so its diagnosis is challenging. Because the treatment of PACNS differs from the treatments required for other CNS diseases, it is vital to make an accurate diagnosis as early as possible.

The diagnosis of PACNS is challenging, and a definite diagnosis can only be made using biopsy findings after brain surgery [7–14] or stereotactic biopsy [15]. However, there are no reports of specialized methods based on radiological features to guide stereotactic biopsy for the diagnosis of PACNS. Here, we report a case with a small PACNS diagnosed with stereotactic biopsy of targets identified by diffusion-weighted magnetic resonance imaging (DW-MRI) and apparent diffusion coefficient (ADC) mapping.

## Case report

A 28-year-old woman who had experienced inactive headache and right limbs numbness with progressive aggravation for 4 days was admitted to our hospital on June 26, 2017. She had experienced no fever since symptom onset. She demonstrated clear consciousness with accurate verbal responses. Physical examinations indicated her right upper limb muscle strength was grade III and her right lower limb muscle strength was grade IV in Manual Muscle Testing Scale. Her right hand could maintain grip, but her fingers had trouble completing delicate motor tasks. There was no clinical history of any relevant CNS, neuronal, or muscles related illness.

Serological tests performed on admission revealed a white blood cell (WBC) of 7.38 × 10^9^/L (normal range 4.0–10.0 × 10^9^/L), percentage of neutrophils of 59.5% (normal range 50–70%), percentage of lymphocytes of 31.3% (normal range 15–70%), percentage of monocytes of 7.6% (normal range 3.0–10.0%), neutrophil count of 4.39 × 10^9^/L (normal range 2.0–7.5 × 10^9^/L), lymphocyte count of 2.31 × 10^9^/L (normal range 0.8–4.0 × 10^9^/L), and monocyte count of 0.56 × 10^9^/L (normal range 0.3–0.8 × 10^9^/L).

**Fig. 1:**
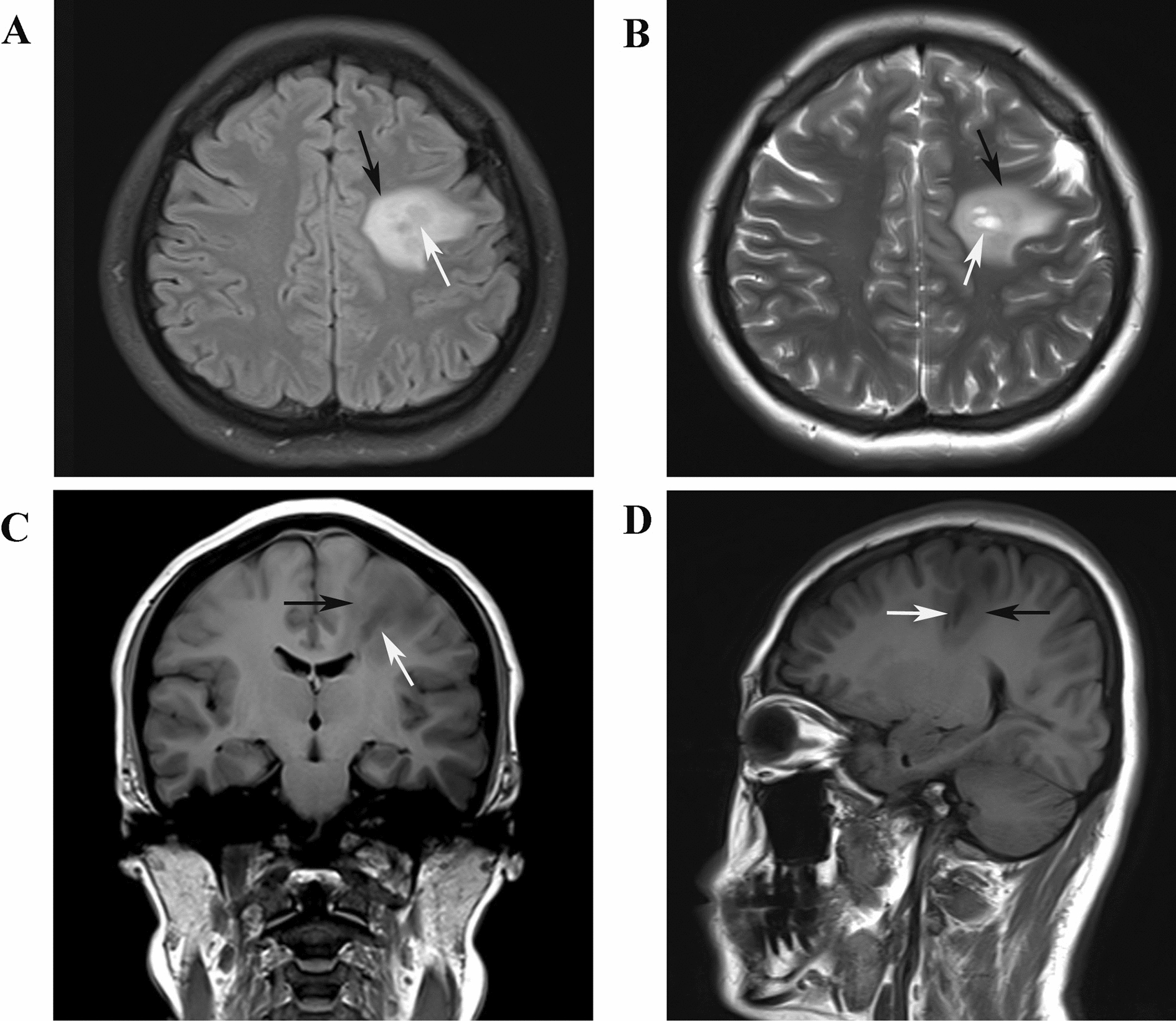
MR images before surgery show an area of inhomogeneous hypointensity on a T1-weighted image (T1WI) and hyperintensity on a T2-weighted image (T2WI) and T1-weighted fluid-attenuated inversion recovery (FLAIR) image, with a size of 15 × 17 × 22 mm^3^ (volume 3.1 cm^3^), surrounded by edema, within the left centrum semiovale (white arrows point to the center of focus; black arrows point to the region of edema). **A** T1-FLAIR axial view. **B** T2WI axial view. **D** T1WI coronal view. **D** T1WI sagittal view

**Fig. 2:**
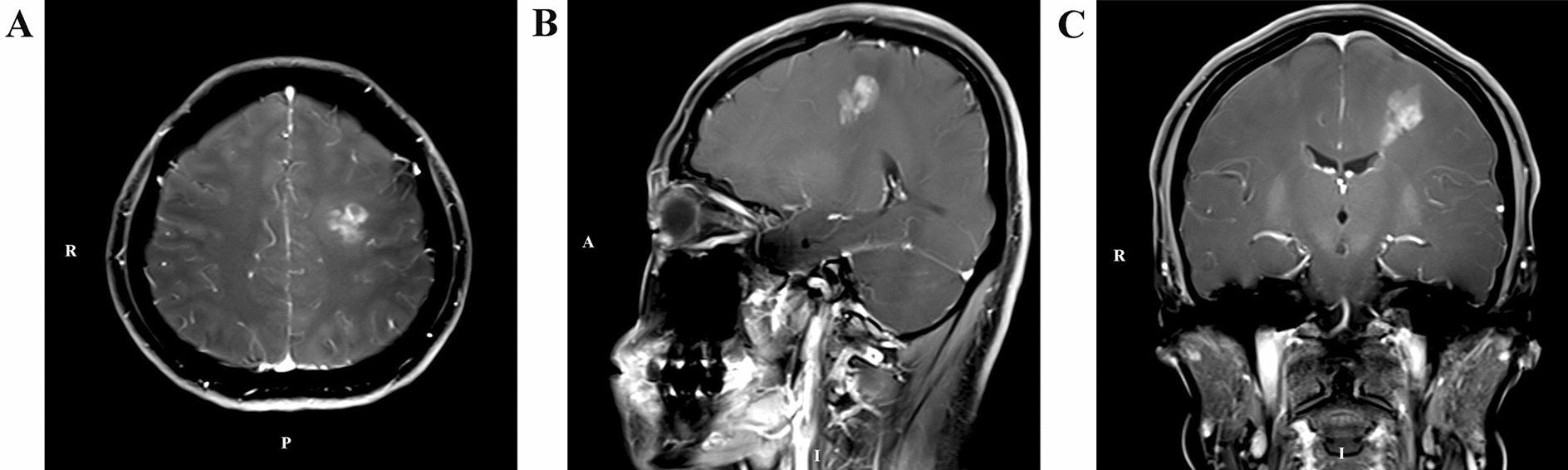
Before surgery, contrast-enhanced T1-weighted magnetic resonance imaging showed a lesion within the left centrum semiovale, with apparently inhomogeneous enhancement and an irregular shape. Edema showed no enhancement. **A** Axial view. **B** Sagittal view. **C** Coronal view

**Fig. 3:**
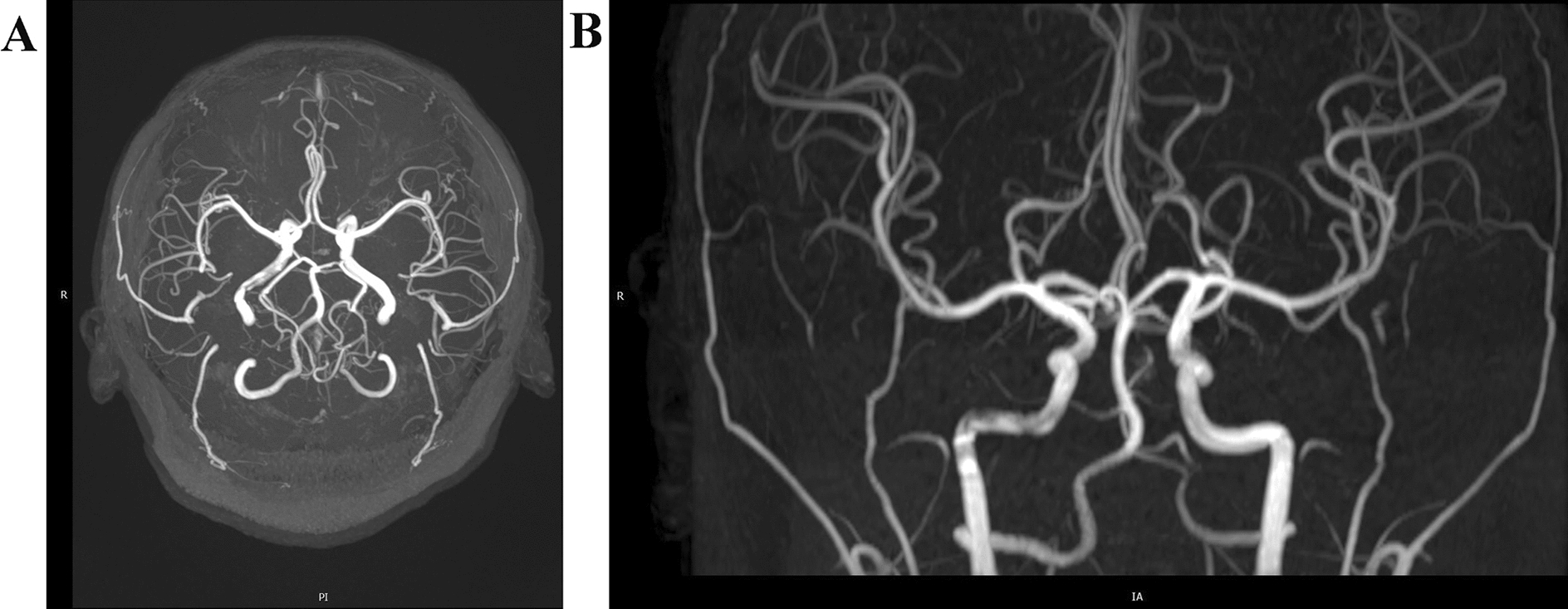
Before surgery, magnetic resonance angiography (MRA) images showed no obvious anomalies. **A** MRA maximum-intensity-projection (MIP) superior image. **B** MRA–MIP anterior image

**Fig. 4:**
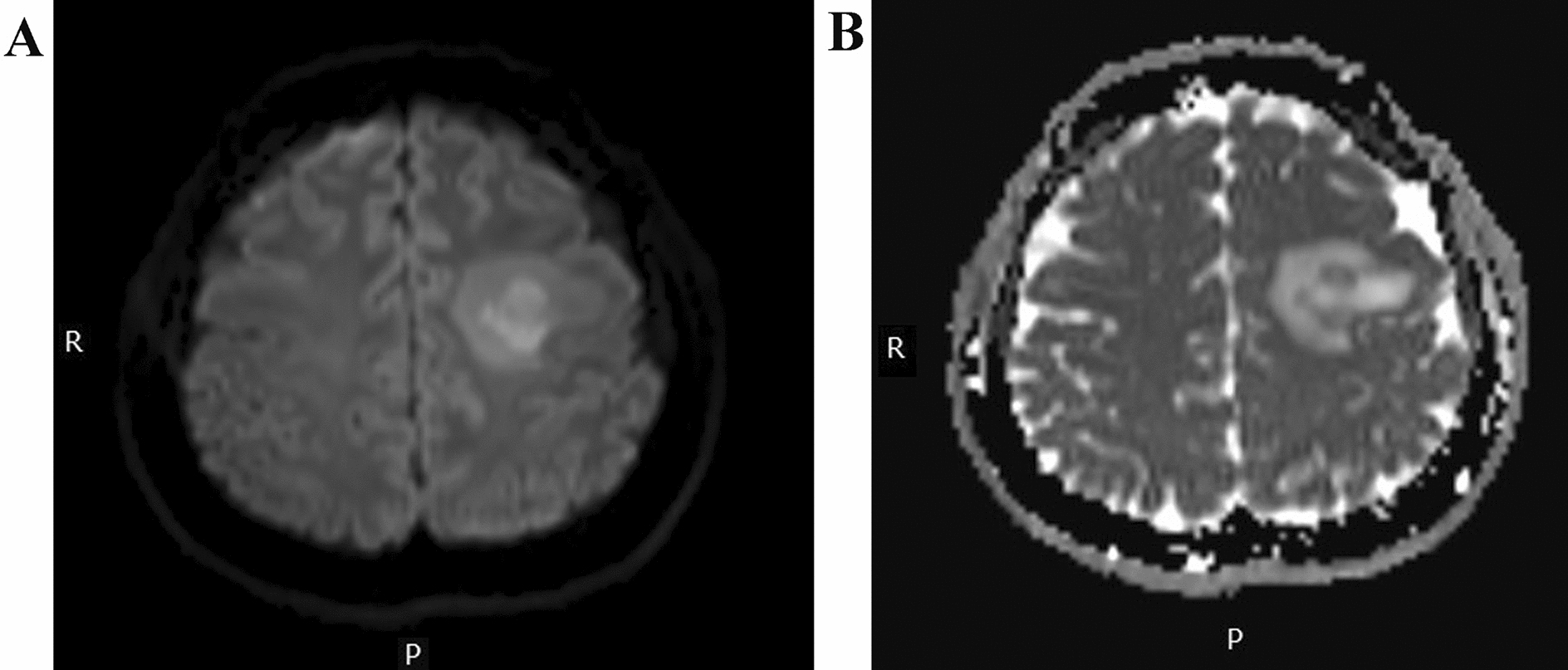
Before surgery, diffusion-weighted magnetic resonance imaging (DW-MRI) and apparent diffusion coefficient (ADC) mapping showed an inhomogeneous hyperintense area within the left centrum semiovale center. DW-MRI revealed a core of hyperintensity, which was hypointense on the ADC map. **A** DWI image (*b* = 1000 s/mm^2^). **B** ADC map

**Fig. 5:**
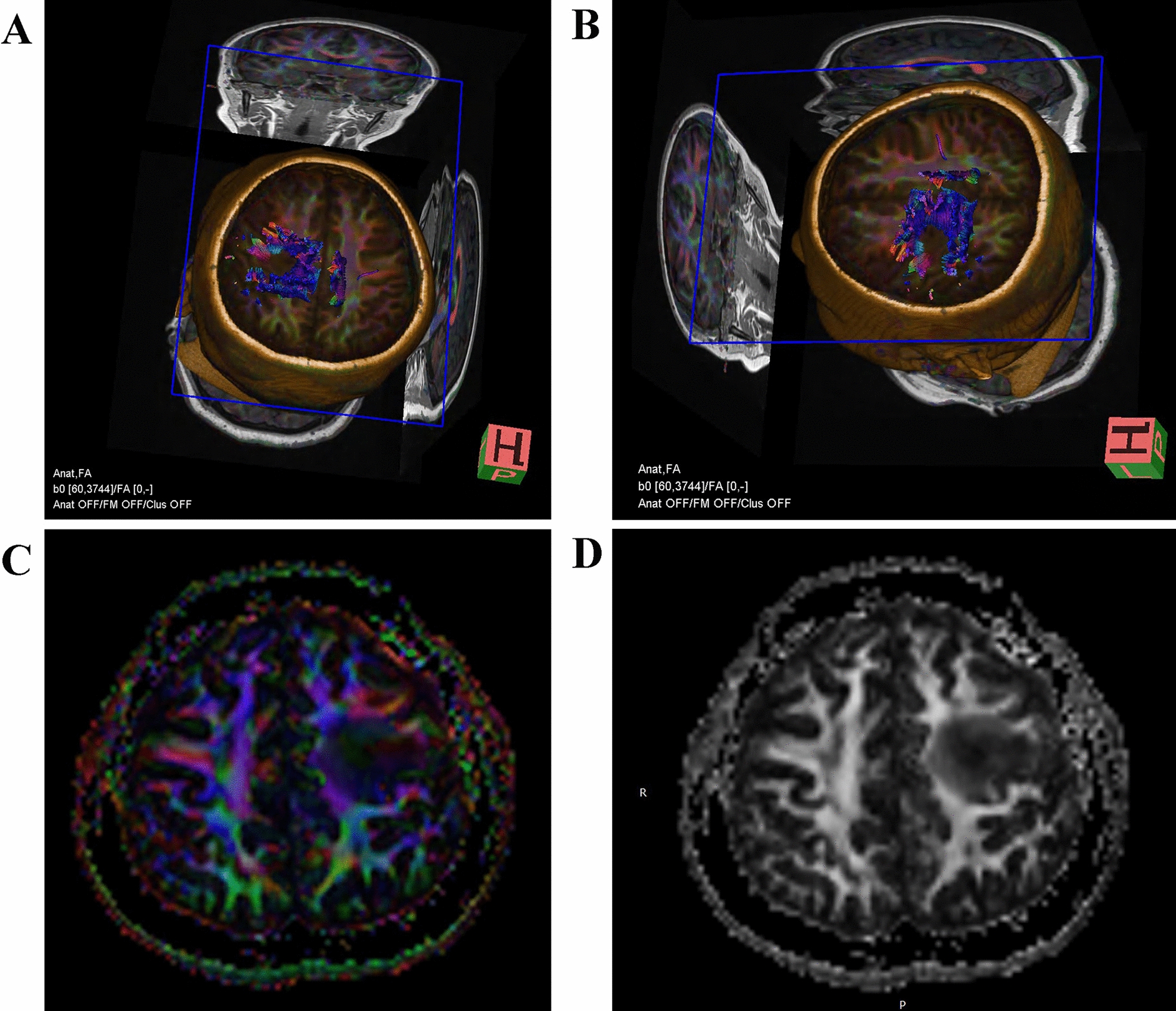
Before surgery, magnetic resonance diffusion tensor images showed that parts of the white matter fiber tracts were interrupted and an area of lower fractional anisotropy (FA) within the left centrum. **A**, **B** Diffusion tensor tractography (DTT). **C** FA pseudocolor image. **D** FA gray-scale image

**Fig. 6:**
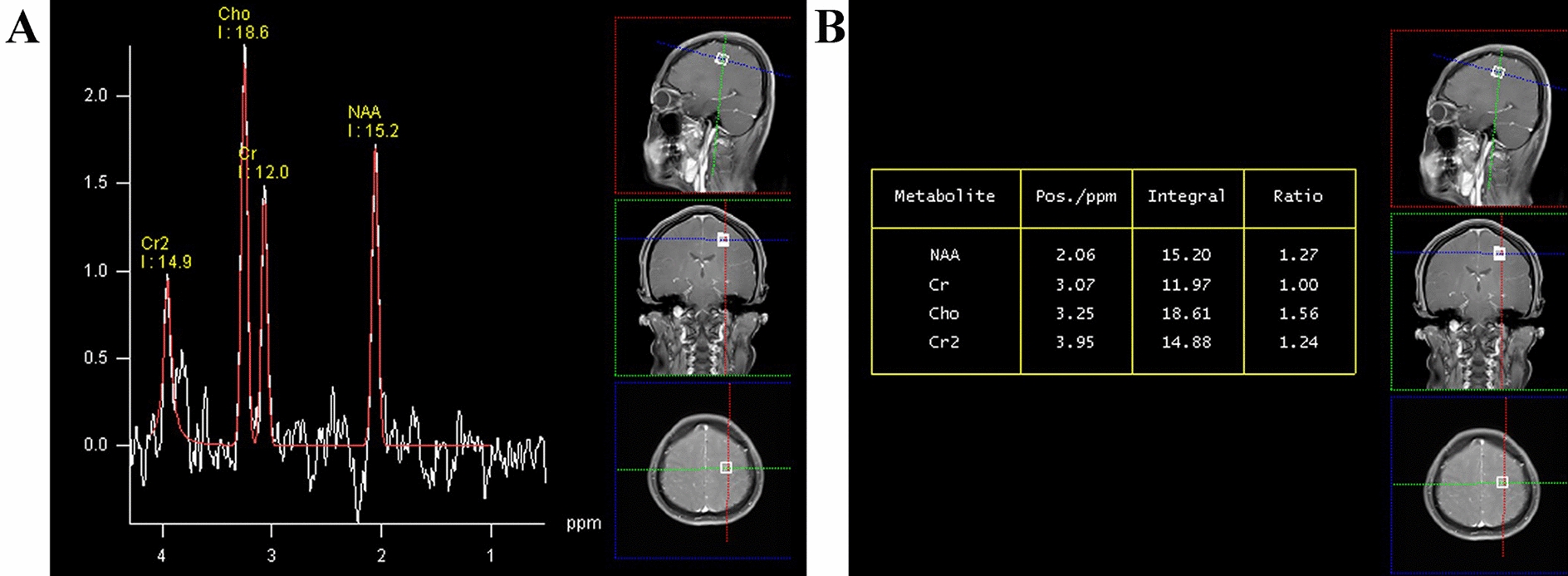
In single-volume spectroscopy–magnetic resonance spectroscopy (SVS–MRS) images before surgery, the MRS content of choline (Cho) and the Cho/creatine (Cr) ratio were increased, whereas the *N*-acetyl aspartate (NAA) content declined slightly within the same area. **A** MRS graph of each metabolite and the locations of each single volume. **B** Table of each metabolite and their ratios to Cr

Two days after admission, MRI showed an area of inhomogeneous hypointense signal on a T1-weighted image (T1WI) and a hyperintense signal on T2-weighted image (T2WI) and T1-weighted fluid-attenuated inversion recovery (FLAIR) images, with a size of 15 × 17 × 22 mm^3^ (volume 3.1 cm^3^), surrounded by edema, within the left centrum semiovale (Fig. 1). A contrast-enhanced MRI (CE-MRI) scan showed a lesion within the left centrum semiovale, with apparent inhomogeneous enhancement and an irregular shape, whereas the surrounding edema showed no enhancement (Fig. 2). Magnetic resonance angiography (MRA) showed no apparent anomalies (Fig. 3). DW-MRI and ADC mapping showed an area of inhomogeneous hyperintensity within the left centrum semiovale. The core of the hyperintense signal on DWI was hypointense on the ADC map (Fig. 4). Magnetic resonance diffusion tensor imaging (DT-MRI) showed that parts of the white matter fiber tracts were interrupted and identified an area of lower fractional anisotropy (FA) within the left centrum semiovale (Fig. 5). Magnetic resonance spectroscopy (MRS) images showed that the content of choline-containing compounds (Cho) and its ratio to creatine (Cr) were increased in this area. In contrast, the content of *N*-acetyl aspartate (NAA) was slightly reduced within the volume in this area: NAA/Cr = 2.54, Cho/Cr = 1.62, Cho/NAA = 1.22 (Fig. 6). The imaging results suggested a strong likelihood of brain glioma.

At day 3 of admission, an analysis of her cerebrospinal fluid (CSF; three tubes were collected, 2 mL per tube) showed colorless CSF with a pressure of 100 mm H_2_O (normal range 80–180 mm H_2_O). The patient’s WBC count was 1 × 10^6^/L (normal range 0–8 × 10^6^/L) and her protein, glucose, and chlorine levels in the CSF were 0.31 g/L (normal range, 0.12–0.60 g/L), 3.40 mmol/L (normal range 2.2–3.9 mmol/L), and 121.4 mmol/L (normal range, 120.0–132.0 mmol/L), respectively. The samples were negative for Pandy’s reaction. CSF cytology revealed no exfoliated cells.

**Fig. 7:**
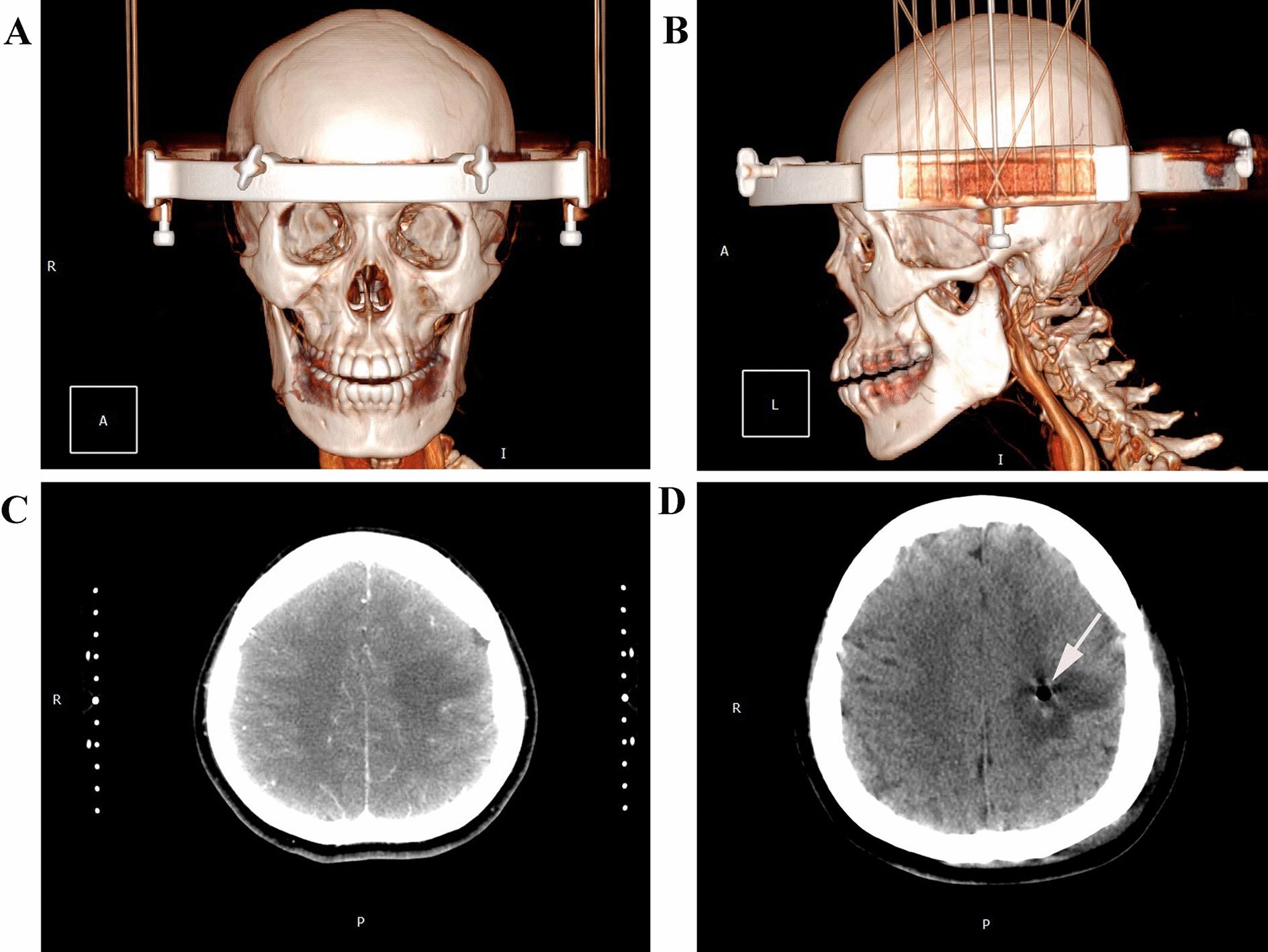
Three-dimensional (3D) computed tomography (CT) images before and after stereotactic biopsy surgery. **A** Localization of the area for stereotactic surgery and an axial image of the 3D CT reconstruction. **B** Stereotactic biopsy localization and sagittal image of the 3D CT reconstruction. **C** Stereotactic biopsy targeting the left frontal lesion. **D** CT scan of the head, 1 day after stereotactic biopsy, showed a shadow indicating gas present in the left frontal lesion (white arrow)

The radiological diagnosis were initially consistent with angiitis, low-grade glioma, or metastatic carcinoma. On day 10, the patient underwent stereotactic biopsy with Komai’s stereotactic instrument (Mizuho Medical Innovation, Tokyo, Japan) (Fig. 7). The area of edema around the lesion in the left centrum semiovale was initially targeted, and 2 × 2 × 2 mm^3^ of white soft tissue was sampled. The second and third biopsies targeted areas on the DWI/ADC map with different characteristics, and sampled two 2 × 2 × 3 mm^3^ regions of gray, slightly toughened tissue. A CT scan of the head performed 1 day after stereotactic surgery showed the presence of gas (evident as a shadow) in the left centrum semiovale (Fig. 7D).

**Fig. 8:**
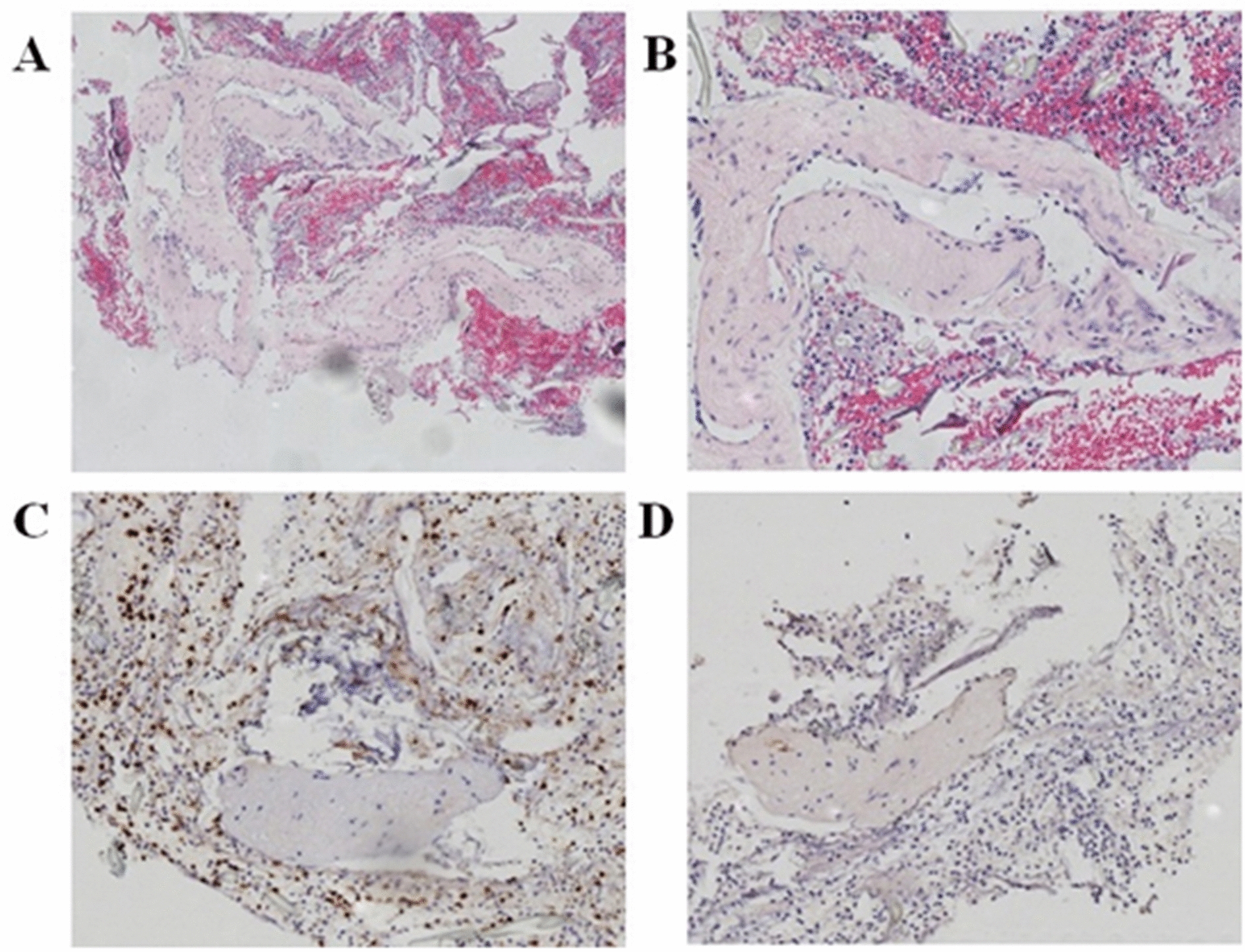
The postoperative pathological diagnosis was vasculitis. Hematoxylin–Eosin staining (HE) shows the histology of isolated vasculitis; immunohistochemistry (IHC) shows the expression of leucocyte common antigen (LCA) and smooth muscle actin (SMA) in isolated vasculitis. **A** HE (40×). **B** HE (100×). **C** IHC of LCA expression (100×). **D** IHC of SMA expression (100×)

Pathological analysis of the biopsies returned a diagnosis of primary angiitis of the central nervous system (Fig. 8). Therefore, she was prescribed an anti-inflammatory medication (cefazolin powder, 2 g twice daily by intravenous injection) and hormone therapy (hydroprednisone, 50 mg twice daily by intravenous injection).

**Fig. 9:**
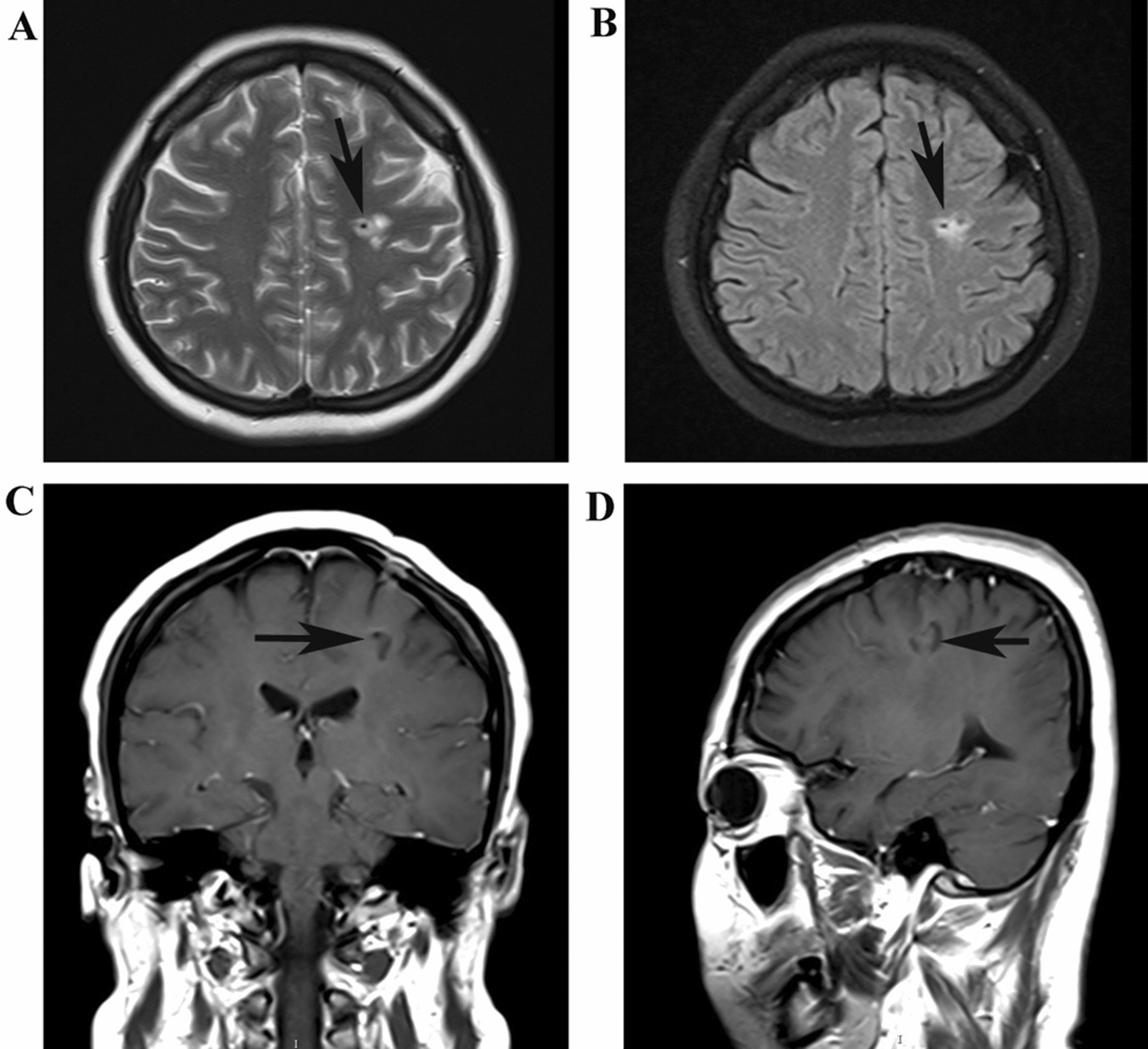
Magnetic resonance images obtained 2 months after surgery showed a small residual cavity of hypointensity on T1-weighted imaging (T1WI) and hyperintensity on T2-weighted imaging (T2WI) and fluid-attenuated inversion recovery (FLAIR), with a clear boundary in the left centrum semiovale after surgical treatment with no enhancement (arrows point to the center of the focus). **A** T2WI axial view. **B** T2-FLAIR axial view. **C** Contrast enhanced (CE)-T1WI coronal view. **D** CE-T1WI sagittal view

Ten days after surgery, the patient’s right upper limb muscle strength was grade IV and her right lower limb muscle strength was grade V in Manual Muscle Testing Scale. At 15 days after surgery, her right upper limb muscle strength was grade V. The patient’s symptoms of limbs have improved significantly. A follow-up MRI examination of the head was conducted 2 months after stereotactic surgery. MRI showed a small residual cavity of hypointensity on T1WI and hyperintensity on T2WI and FLAIR with a clear boundary in the left centrum semiovale after surgical treatment, with no enhancement (Fig. 9). A follow-up MRI examination of the head 18 months after stereotactic surgery showed that the small cavity in the left centrum semiovale had disappeared. The 3-year follow-up confirmed that the patient had recovered well, with a Glasgow Outcome Scale score of five.

## Discussion

The methods used to diagnose PACNS primarily include CSF examination, imaging, and pathological investigation. Pathology plays a vital role in the diagnosis of PACNS, whereas imaging plays an auxiliary role to the pathological examination. If no pathological examination is made, PACNS can only be an exclusionary diagnosis [1, 6, 15].

Imaging is extremely valuable in the diagnosis of PACNS. As well as the traditional technologies of CT, MRI, and cerebrovascular imaging, such as computed tomography angiography (CTA), MRA, and digital subtraction angiography (DSA), the diagnosis of PACNS with various other imaging techniques has been reported, including new molecular imaging procedures [16–18]. Most PACNS patients have various MRI abnormalities [19], but some PACNS patients have normal angiograms [20]. MRA and DSA examinations of PACNS patients can detect stenosis or dilation of multisegment blood vessels in ischemic lesions, but rarely find long, complete vascular occlusions or aneurysm-like changes [1, 6]. Until recently, DSA was the most widely used technique to diagnose PACNS [6, 18, 21]. The most typical manifestations of PACNS on MRI are multiple asymmetric ischemic lesions involving the bilateral cerebral hemispheric cortex, subcortical structure, and deep white matter, accompanied by intracerebral or subarachnoid hemorrhage. Other rare manifestations include hemorrhage-like, mass-like, or cyst-like lesions in the brain parenchyma, which are challenging to distinguish from other cerebrovascular diseases, demyelination diseases, and brain tumors [6, 22]. Patients with a solitary mass-like PACNS are relatively rare (< 5%), and the differential diagnosis is more difficult [1]. The literature also reports that DWI and DTI examinations can detect PACNS patients with negative MRI results [6, 23], and DWI sequences can detect acute ischemic lesions [21]. In study a 16 patients, 15 showed DWI hyperintensity and nine of these also showed hypointensity in the middle of the lesion. All 16 patients underwent a brain biopsy (a stereotactic procedure in 10 and open-wedge surgery in six), which showed lymphocytic angiitis in the majority of patients (15/16) and necrotizing angiitis in one patient [24]. In one patient with PACNS with a tumor-like mass lesion, there was no evidence of acute infarction on DWI [25].

Tissue biopsy is the key to diagnosing PACNS [26], and the role of pathological biopsy in the diagnosis of PACNS is irreplaceable. Brain biopsy is now considered the ‘gold standard’ strategy for the definitive diagnosis of PACNS [19] and should be pursued not only because it provides information that establishes the diagnosis but also effectively excludes other similar diseases [7, 20, 21, 27]. Imaging methods cannot detect some cases of PACNS involving peripheral blood vessels. However, even when imaging reveals typical PACNS manifestations, the biopsy results may not support PACNS diagnose [6]. Pathology results confirm that the inflammation associated with PACNS usually manifests as transmural damage to the involved vessel wall and immune cell infiltration. Thickening of the vessel wall can cause vascular stenosis, disturbing the microcirculation. Simultaneous rupture of the vessel wall and secondary intracranial hemorrhage can occur due to the fragility of the vessel wall [6].

Areas of CT or MR enhancement, or areas of abnormal MRS are often been used to guide stereoscopic biopsy and localize intracranial lesions. A framed stereotactic device biopsy, positioning by CT-enhanced, MR-enhanced T1, T2-weighted imaging and MRA-enhanced imaging, was used to diagnose a case of PACNS [15]. The diagnosis rate can reach 80% when imaging is used to assist pathological biopsy [6, 22]. In one study, the classic angiographic features of angiitis were associated with biopsy or postmortem confirmation in only 4.6% of patients [28] (32/701), and positive angiography was not correlated with positive biopsy results in a study of 34 PACNS patients [29]. The diagnosis rate of PACNS achieved with surgical biopsy ranged from 36 to 83% in previous studies [13, 15].

The surgical biopsy complication rate is 16%, which includes serious complications such as cerebral hemorrhage and epilepsy [13, 15], whereas stereotactic biopsy is less traumatic. It can be used in critically ill patients, and its complication rate is < 13%, with patients experiencing only transient or minor complications [15]. Therefore, the diagnosis rate of pathological biopsy should be increased as possible for the accurate diagnosis and appropriate treatment of patients with PACNS. Because the number of cases is small and clinical experience of this disease is limited, the process of PACNS biopsy has not been standardized. The leptomeninges, cortex, and deep white matter must be sampled simultaneously to improve the diagnostic biopsy rate [13, 15].

The low incidence of PACNS and its high heterogeneity means that there is a lack of randomized controlled trials of its treatment [6]. Glucocorticoids alone or in combination with cyclophosphamide can achieve satisfactory results [22]. However, it was reported that the administration of azathioprine, methotrexate, or mycophenolate mofetil as maintenance therapies can achieve more satisfactory results [27].

Our patient had a small intracranial lesion (with a volume of 3.1 cm^3^). MRI and MRA examinations showed that the left vertebral artery was narrow, consistent with the imaging features reported in the literature [23]. Limaye et al. reported that when two patients (20%) underwent MRS, the Cho/NAA ratios in both patients were approximately 0.9 [19]. The result of MRS in this patient (Cho/NAA = 1.22) does not have a noticeable prompting effect on the judgment of the lesion's nature. We considered that, because the MR T2 and FLAIR features of PACNS patients can be either normal or abnormal, they could have abnormal DWI and ADC images. Therefore, we performed comprehensive imaging examinations before performing stereotactic biopsy in our patient. The imaging examination allowed areas with different characteristics on the DWI and ADC maps to be selected as multiple targets for stereotactic biopsy, which allowed us to make a precise pathological diagnosis. Follow-up for 3 years confirmed that the patient recovered well after treatment (Glasgow Outcome Scale of 5).

## Conclusion

The diagnosis and treatment of this patient suggest that for patients suspected of PACNS, presetting the puncture targets for stereotactic biopsy according to various features on DW-MRI and ADC maps will improve the diagnosis rate of diagnostic biopsy surgery. Imagings assist target localization, and can used to support stereotactic biopsy for the diagnosis of small intracranial lesions in patients with PACNS.

## Data Availability

Not applicable.

## Abbreviations

MR: Magnetic resonance
MR-DWI: Diffusion-weighted magnetic resonance imaging
ADC: Apparent diffusion coefficient image
DTI: Diffusion tensor imaging
1H-MRS: Magnetic resonance spectroscopy
CT: Computed tomography
GOS: Glasgow Outcome Scale
PACNS: Primary angiitis of the central nervous system
WBC: White blood cell
MR CE: Contrast enhanced magnetic resonance
MRA: Magnetic resonance angiography
FA: Fractional anisotropy
Cho: Choline-containing compounds
Cr: Creatine
NAA: *N*-acetyl aspartate
CSF: Cerebrospinal fluid
CTA: Computed tomography angiography
DSA: Digital subtraction angiography
SAH: Subarachnoid hemorrhage
